# COVID-19 Booster Uptake among First Responders and Their Household Members May Be Lower than Anticipated

**DOI:** 10.3390/vaccines10071011

**Published:** 2022-06-24

**Authors:** Jennifer A. Frey, Daniel J. Bachmann, Mirela Anghelina, Valerie Sircelj, Osama Saadoon, Patrick Stevens, Maciej Pietrzak, Soledad Fernández, Ann Scheck McAlearney, Ashish R. Panchal

**Affiliations:** 1 Department of Emergency Medicine, The Ohio State University Wexner Medical Center, Columbus, OH 43210, USA; jennifer.frey2@osumc.edu (J.A.F.); daniel.bachmann@osumc.edu (D.J.B.); valerie.sircelj@osumc.edu (V.S.); osama.saadoon@osumc.edu (O.S.); 2 Center to STOP-COVID, The Ohio State University, Columbus, OH 43210, USA; ann.mcalearney@osumc.edu; 3 Department of Biomedical Informatics, College of Medicine, The Ohio State University, Columbus, OH 43210, USA; mirela.anghelina@osumc.edu (M.A.); patrick.stevens@osumce.edu (P.S.); maciej.pietrzak@osumc.edu (M.P.); soledad.fernandez@osumc.edu (S.F.); 4 Center for Biostatistics, College of Medicine, The Ohio State University, Columbus, OH 43210, USA; 5 The Center for the Advancement of Team Science, Analytics, and Systems Thinking in Health Services and Implementation Science Research (CATALYST), College of Medicine, The Ohio State University, Columbus, OH 43210, USA; 6 Department of Family and Community Medicine, College of Medicine, The Ohio State University, Columbus, OH 43210, USA

**Keywords:** COVID-19, vaccination, booster, first responder, frontline healthcare workers, emergency medical services, households

## Abstract

(1) Background: COVID-19 vaccination status varies widely among law enforcement and emergency medical services professionals. Though at high risk of exposure, these first responders have demonstrated significant vaccine hesitancy, with only 70% reportedly vaccinated. We sought to understand whether similar vaccine hesitancy exists for first responders and their household contacts around COVID-19 boosters. (2) Methods: In a prospective longitudinal cohort of first responders and their household contacts, survey data was collected, including demographics, medical history, COVID-19 exposure risks, and vaccination and/or booster status. The statistical analysis focused on primary vaccination and booster rates of both the first responders and their household contacts. (3) Results: Across 119 study participants, 73% reported having received some combination of vaccine and/or booster, and 26% were unvaccinated. Vaccinated individuals were older, reported less prior exposure to COVID-19 and had more comorbidities. Only 23% reported having received a COVID-19 booster. Pairing of the data for household contacts demonstrated a 60% agreement to receive primary vaccination but only a 20% agreement for boosters within households. (4) Conclusions: This study provides insight into the vaccination and booster rates of first responders and household contacts. Focused efforts to enhance vaccinations is essential for the protection and maintenance of this critical workforce.

## 1. Introduction

Throughout the COVID-19 pandemic, first responders have been on the frontlines of the medical response [1]. As the first contact for emergency medical care in many areas globally, these providers have therefore been at an increased risk for infection and illness [2,3]. Risk reduction for these first responders through vaccinations is critical for both their own safety and the safety of the patients they treat.

Unfortunately, significant hesitancy to receive COVID-19 vaccinations among the emergency medical services (EMS) profession has been shown prior to and after the release of the vaccine [4,5,6,7]. Some of the drivers for these decisions focus on first responders’ lack of confidence in the safety and rapid development of the vaccines, their perceived risk of COVID-19 exposure and infection, and a general distrust of medical care system [7,8].

The COVID-19 vaccination strategy has recently been enhanced with the addition of COVID-19 boosters, and a combination of vaccinations with boosters has demonstrated efficacy in protecting individuals from new strains of COVID-19 [9,10,11]. It is unclear, however, whether similar vaccination hesitancy exists for first responders towards boosters as was demonstrated with initial vaccinations. Additionally, there is no information on whether the household members of first responders are similarly vaccinated as the providers themselves, which is a critical knowledge gap since exposures can occur from within the household.

As vaccinations and subsequent COVID-19 boosters are now readily available in the US, we sought to improve our understanding about the vaccination choices in a longitudinal cohort of first responders as well as their household contacts. This insight can be used to help shape long-term interventions designed to increase the uptake of vaccinations and boosters and thus, bolster protection in this important population of healthcare providers that is particularly exposed to COVID-19.

## 2. Subjects and Methods

### 2.1. Study Design and Setting

This is a prospective longitudinal cohort study of first responders and their household contacts in Columbus, Ohio. This study is part of a larger effort of a U54 grant [#U54CA260582] led by the Center for Serological Testing to Improve Outcomes from Pandemic COVID-19 (STOP-COVID), which has an overarching goal of understanding the prevalence and transmission of COVID-19. The funders of the study had no role in the design of the study protocol, data collection, data management, data analysis, data interpretation, or writing of the manuscript. The Institutional Review Board approved the protocol (IRB#2020H0531) and informed consent was obtained from all participants in this study.

### 2.2. Study Design Population

First responders and their household contacts were recruited to be part of a longitudinal study to understand COVID-19 and immunity. Participants were 18 years or older, a first responder (police/fire/EMS) or lived in the household of one, were willing to have COVID-19 clinical tests and blood collection minimally twice a year, and were willing to participate in brief recurring surveys about demographics, health risks, and exposures. Participants were recruited through fliers distributed at their respective agencies (i.e., fire, police) from January 2021–November 2021.

### 2.3. Measurements

Following enrollment, participants completed surveys concerning personal demographics and medical history, including COVID-19 vaccination status. Demographic information included age, gender (male, female), and race (White, Black, Asian or Pacific islander, American Indian, other, unknown). Medical history included whether participants had been previously infected with COVID-19, smoking status (current or history of smoking/vaping), and past medical history focusing on the diseases included in the Charlson Comorbidity Index [12]. Vaccination status was collected and confirmed at least every 6 months with participants; this was defined as unvaccinated, receipt of first dose of COVID-19 vaccine, receipt of first/second dose of COVID-19 vaccine without booster (full vaccination), or receipt of full COVID-19 vaccine plus booster. Type of vaccine received (e.g., Moderna, Pfizer, etc.) was also collected.

Household contacts were defined as individuals living together in the same home at least 50% of the time. Unique household identifiers were generated for each first responder and their associated household contacts.

### 2.4. Statistical Analysis

The cohort was evaluated based on vaccination status defined as either unvaccinated or vaccinated (received at least 1 vaccination). Descriptive statistics and two-group comparisons (*T*-test, Chi-square or Fisher’s exact test depending on the outcome) were performed. All enrolled participants were included. For those variables that had missing data, the total number of participants was assumed as the denominator for consistency across proportions or means. For the primary analysis, household pairs were coded as to whether they disagreed or agreed (both for and against) with respect to receiving a COVID-19 vaccine. The differences of the three proportions between the groups: unvaccinated, COVID-19 full vaccine, or booster were compared using Fisher’s exact test. The difference in agreement on COVID-19 full vaccine or booster within households was tested using McNemar’s test to account for the correlations between individuals in the same household. All analyses were completed using STATA SE version16 (StataCorp LP, College Station, TX, USA).

## 3. Results

The study enrolled 119 participants by November 2021, and the demographics of this population are presented in Table 1. The unvaccinated and vaccinated populations had similar distributions by gender and race. The vaccinated group contained more participants who were older, had fewer reported COVID-19 infections, had more comorbidities, and had more current or past smokers.

Table 2 shows the vaccination rates for the cohort. Table 2 shows the vaccination rates for the cohort. In this population, 26% were unvaccinated, and 67% of participants received full primary vaccination (with or without booster). Full primary vaccine plus booster rate for the cohort was only 23%. Vaccination with a Moderna vaccine was most common across this cohort, followed by vaccination with a Pfizer vaccine.

**Figure 1:**
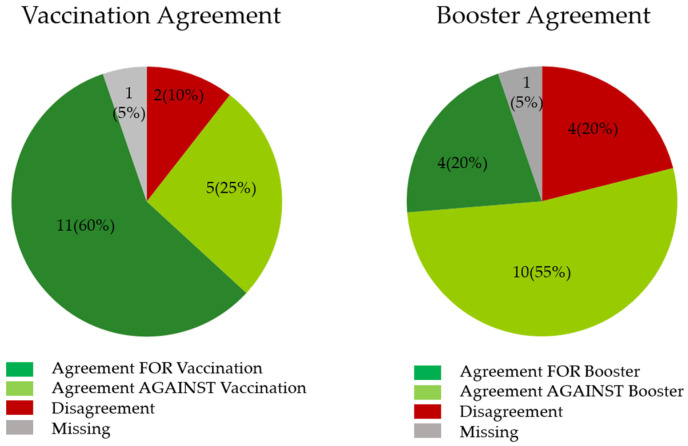
Agreement within households was different for receiving primary COVID-19 vaccinations and boosters (*p* < 0.005). Agreement for primary vaccination was high (60%), whereas agreement for boosters was low (20%) within households.

Across the cohort, there were 19 total households identified to have more than 1 study participant. When evaluating the agreement between the first responder and their household contacts around the receipt of a COVID-19 vaccine, we noted differences in agreement around receiving both primary vaccines and boosters for COVID-19 (*p* < 0.005) (Figure 1). With respect to primary vaccination, agreement within the household for receipt of the vaccine was 60%. In contrast, the agreement against receipt of the booster was high (55%), with only 20% agreement for receiving a booster shot. Household disagreement around boosters was higher at 20%. There was a low rate of disagreement for both primary full vaccinations and boosters within households with respect to decisions about vaccination.

## 4. Discussion

Through the evolving pandemic, one of the key challenges to improving patient outcomes has been consistent vaccinations across all populations. Unfortunately, even in populations considered to be at a high risk of exposure, such as first responders, consistent high rates of vaccinations have been difficult to achieve [13,14]. Much of this has been due to vaccine hesitancy, and this hesitancy has been shown to be driven by many factors [15]. Fundamentally, as we transition from primary vaccinations to the need for boosters to manage evolving strains of COVID-19, understanding the true acceptance of boosters will be critical.

In this cohort of first responders and their associated household contacts, 72% were at least partially COVID-19 vaccinated, but only 23% of study participants reported having received a COVID-19 booster. Household members’ decisions about primary vaccination were significantly aligned (60%). However, there was also a high level of agreement against the receipt of COVID-19 boosters (55%). The reasons behind this difference are unclear and will require further evaluation to understand whether the drivers of vaccine hesitancy are similar for booster hesitancy.

This evaluation of first responders and their household contacts during the COVID-19 pandemic is particularly important since transmission may occur by exposure when providing patient care or through their household, and when conducting other daily activities. Significant concerns early in the pandemic for these front-line providers included shortages of personal protective equipment (PPE), long exposure times, and inadequate training, which contributed to COVID-19 exposure for healthcare workers [16,17]. As the pandemic has progressed and evolved, when sufficient PPE was available, exposure was shown to occur primarily outside the provision of direct clinical care [18,19,20,21]. Specifically, an evaluation of first responder COVID-19 infections from February 2020–July 2020, with ample availability of PPE for clinical care, demonstrated overall prevalence to be 0.57 infections/10,000 person-days; exposures originated primarily from non-patient care sources [18]. Following households and understanding the choices made within households concerning vaccinations will be critical for future pandemic preparedness efforts, as well as determining appropriate messaging to optimize vaccination efforts.

This study is limited in several ways. This population is a small cohort of first responders enrolled in a longitudinal study, making this a convenience sample. This suggests that the population may not be representative of the national opinions concerning vaccinations in first responders. Selection bias (consent bias) of our enrolled population should also be considered due to voluntarily participation in the study. In our cohort, the consent rate was 66%, with 119 participants enrolled out of 180 people contacted by our research team. The amount of consent bias cannot be estimated because the subjects who did not consent remained unobserved, and the majority did not give a reason for their decision not to participate. However, the results and demographics of this study population are similar to nationally representative study samples of first responders (e.g., age, gender, race) [8,22]. Furthermore, this study demonstrates similar overall vaccination rates as noted in a nationally representative sample of EMS professionals at 70% [8]. This data adds credence to the findings and concerns we have identified in this study.

## 5. Conclusions

In this cohort of first responders and their associated household contacts, 72% were at least partially COVID-19 vaccinated, but only 23% of study participants reported having received a COVID-19 booster. This study suggests that COVID-19 booster uptake among first responders and their household members may be lower than anticipated. Focused efforts to enhance vaccinations is essential for the protection and maintenance of this critical workforce.

## Figures and Tables

**Table 1:** Cohort demographics by vaccination status.

	Full Cohort (*n* = 119)	Unvaccinated (*n* = 31)	Vaccinated (*n* = 86)	*p* Value
Age				
Median, IQR	47 (38–52)	38.5 (31–50.5)	48 (41–52)	0.002
Gender (*n*, %)				
Male	76 (63.9)	18 (58.1)	58 (67.4)	0.7
Female	37 (31.1)	10 (32.3)	27 (31.4)	
Unknown	6 (5.0)	3 (9.7)	1 (1.2)	
Race (*n*, %)				
White	102 (85.7)	24 (77.4)	78 (90.7)	0.4
Black	6 (5.0)	3 (9.7)	3 (3.5)	
Other	3 (2.5)	1 (3.2)	2 (2.3)	
Asian or Pacific Islander	0 (0.0)	0 (0.0)	0 (0.0)	
American Indian	1 (0.8)	0 (0.0)	1 (1.2)	
Unknown	7 (5.9)	3 (9.7)	2 (2.3)	
Previous SARS-CoV-2 infection (*n*, %)				
Yes	41 (34.5)	13 (41.94)	28 (32.6)	<0.001
No	45 (37.8)	3 (9.7)	42 (48.8)	
I don’t know	26 (21.9)	12 (38.7)	14 (16.3)	
No response	7 (5.9)	3 (9.7)	2 (2.3)	
Any Comorbidity	48 (40.3)	9 (29.0)	39 (45.4)	0.2
Common Comorbidities				
Hypertension (high blood pressure)	20 (16.8)	2 (6.5)	18 (21.0)	
Asthma	7 (5.9)	1 (3.2)	6 (7.0)	
Other autoimmune diseases	7 (5.9)	1 (3.2)	6 (7.0)	
Cancer—current or past	6 (5.04)	1 (3.2)	5 (5.8)	
Inflammatory bowel disease	5 (4.2)	1 (3.2)	4 (4.7)	
Diabetes (any type)	3 (2.5)	1 (3.2)	2 (2.3)	
Smoking Status				
Never smoked	103 (86.55)	27 (87.1)	76 (88.37)	0.001
Current or former smoker	10 (8.4)	1 (3.23)	9 (10.47)	
Missing	6 (5.04)	3 (9.68)	1 (1.16)	

**Table 2:** Vaccination information for the complete cohort (*n* = 119).

Vaccine Information	*n* (%)
Vaccination	
Unvaccinated	31 (26.1)
Partial primary COVID-19 vaccine	8 (6.72)
Full primary COVID-19 vaccine	51 (42.9)
Full primary COVID-19 vaccine plus booster	27 (22.7)
Unknown	2 (1.7)
Type of Vaccination	
Moderna (2 dose for full primary vaccination)	44 (51.2)
BioNTech, Pfizer (2 dose for full primary vaccination)	36 (41.9)
AstraZeneca (2 dose for full primary vaccination)	1 (1.2)
Johnson and Johnson (1 dose for full primary vaccination)	5 (5.8)

## Data Availability

Not applicable.
